# Influence of Social Support and Quality of Life on Mortality Among Patients Undergoing Hemodialysis

**DOI:** 10.1097/jnr.0000000000000732

**Published:** 2026-03-11

**Authors:** Lu ZHANG, Yongai ZHANG, Sumei ZHANG, Xuanbing TANG, Shengyan SHI, Haiying QUAN, Xiu YANG

**Affiliations:** 1 School of Nursing and Rehabilitation, Xi’an Medical University, Shaanxi, PRC

**Keywords:** hemodialysis, social support, quality of life, all-cause mortality

## Abstract

**Background:**

Although quality of life and social support are widely recognized as positive factors promoting health, few follow-up studies analyzing the predictive significance of social support and quality of life on the prognosis of patients undergoing maintenance hemodialysis have been published.

**Purpose:**

This study was designed to investigate the predictive value of social support and quality of life on all-cause mortality in patients undergoing maintenance hemodialysis.

**Methods:**

The Social Support Rating Scale and Kidney Disease Quality of Life Short-Form 1.3 were used to evaluate social support and quality of life, respectively. Cox analysis was used to analyze the predictive value of social support and quality of life on all-cause mortality.

**Results:**

Over the course of this 3-year follow-up study, all-cause mortality was 30.93%. In terms of quality of life, significant between-group differences were found for two indices in the physical component summary (physical functioning and role-physical), three indices in the mental component summary (social functioning, mental health, and vitality), and two indices in the kidney disease domain (effect of kidney disease and patient satisfaction). Moreover, the surviving patient group reported higher scores on the physical component summary, mental component summary, and 36-item short-form health survey than the deceased patient group. In terms of social support, the surviving patient group earned significantly higher scores for utilization of social support and the social support rating scale total score than the deceased patient group. Based on Cox proportional hazards model results, 36-item short-form health survey and social support rating scale scores were significantly predictive of all-cause mortality.

**Conclusions/Implications for Practice:**

The findings of this study demonstrate the predictive value of social support and quality of life for all-cause mortality in patients undergoing hemodialysis. Healthcare providers should consider addressing social support and quality of life in the care of patients on hemodialysis to improve outcomes and enhance overall well-being in these individuals.

## Introduction

With the advancement of dialysis treatment technologies, the survival rate among patients undergoing maintenance hemodialysis (MHD) has increased. Unfortunately, despite improving hemodialysis technologies, patients undergoing MHD still experience higher rates of hospitalization and mortality than the general population (Johansen et al., 2022). As the number of patients undergoing MHD continues to rise, the demand for related health management and healthcare services has also increased to improve prognoses (Msaad et al., 2019).

Good quality of life (QOL) and social support contribute to promoting health. The results of some studies have confirmed social support and QOL as related to good treatment adherence, thereby affecting chronic disease progression and individual health status (Hermsen et al., 2017; Yang et al., 2020). Furthermore, QOL and social support have been shown to promote the formation of healthy behaviors, control the progression of chronic diseases, regulate balance, and ultimately improve prognosis and promote patient health (Cirillo et al., 2021).

Encompassing systemic and nonmedical factors affecting physical and mental health across the lifespan, social support impacts human health significantly. The social support available to patients on dialysis is generally relatively limited due to the impacts of their disease and dialysis treatment. A significant association between social factors and, respectively, the health management and outcomes of patients with chronic kidney disease (CKD) has been shown, including disease monitoring, grading management of early CKD, kidney replacement therapy, and end-of-life care (Luyckx et al., 2021). Moreover, related studies have highlighted a significant correlation between perceived social support and outcomes, with higher social support scores indicating milder physiological symptoms such as fatigue and pain, as well as milder psychological symptoms such as depression and anxiety (Erickson et al., 2024). However, few follow-up studies have been conducted to analyze the predictive significance of social support on the prognosis of patients with end-stage renal disease, including those on maintenance hemodialysis. Therefore, the main objective of this study was to determine the value of social support factors in predicting mortality risk in patients on MHD using a 3-year follow-up study.

Symptoms such as hypotension, itching, pain, and insomnia widely affect the daily activities of patients undergoing hemodialysis and seriously impact their physiological and psychological functions, thereby affecting their QOL in terms of physiology, psychology, and social interaction (Jesus et al., 2019). QOL is a useful indicator for predicting hospitalization and mortality in patients with CKD and end-stage kidney disease who require renal replacement therapy (Hussien et al., 2021). The Kidney Disease Quality of Life Short-Form 1.3 (KDQOL-SF 1.3) is an important evaluation scale for assessing QOL in patients undergoing hemodialysis. In this study, the KDQOL-SF 1.3 was applied to evaluate the QOL of patients undergoing hemodialysis in both the physiological and psychological domains. In addition, this scale was also used to compare differences in QOL between the deceased and surviving patient groups at baseline. The findings of this study are expected to further clarify scholarly understanding of the predictive function of QOL and social support on all-cause mortality.

## Methods

### Design

For this prospective, observational study, outpatients undergoing treatment at maintenance hemodialysis centers in two hospitals were recruited as participants. Baseline data were gathered between April and May 2021. The outcome event was all-cause mortality, and survival time (in months) was calculated as the time from study enrollment until the outcome event (death) or the final follow-up. The final follow-up for all participants was conducted on April 30, 2024.

### Study Setting and Sampling

A convenience sampling method was used in participant recruitment, and the following formula was used to calculate the minimal sample size required for the proportional risk model (Hsieh & Lavori, 2000):


$$N=\frac{{\left(Z_{1-\alpha /2}+Z_{1-\beta }\right)}^{2}}{P(1-R^{2})\sigma^{2}B^{2}}.$$

Based on the latest research, preliminary experiments, and preliminary research data of the project applicant, the minimum required sample size was estimated to be 108 patients. However, due to the prospective nature of this study and the expected loss of patients over the 3-year follow-up period, a dropout rate of 28% was estimated, requiring a minimum sample size of 139 patients at baseline. Ultimately, valid data on 236 participants were collected and analyzed in this study.

### Inclusion and/or Exclusion Criteria

Data on patients receiving MHD at two hospitals were collected and used in this study. The participant inclusion criteria were (1) on MHD for at least 3 months and (2) over 18 years old. The exclusion criteria were (1) having a diagnosis of cancer, acute infection, or severe heart disease; (2) current hospitalization; (3) unable to cooperate with the study due to mental illness, visual and hearing impairments, or other conditions. All of the participants provided written informed consent.

### Participant Sociodemographic and Medical Variables

Sociodemographic data, including age, gender, educational level, marital status, employment status, primary renal diagnosis, blood pressure, and vascular access status, were collected via face-to-face conversations and a search of patient electronic medical records. Blood pressure (BP) was measured before commencing dialysis sessions.

### Evaluation of Quality of Life

The Chinese version of the Kidney Disease Quality of Life Short-Form 1.3 (KDQOL-SF 1.3; Chow & Tam, 2014) was used in this study to evaluate QOL in two parts, with the first evaluating QOL from 11 kidney disease-targeted areas and the second using a 36-item short-form health survey (SF-36) to evaluate overall QOL in eight areas. The 11 kidney disease-targeted areas are symptoms/problems, effects of kidney disease, burden of kidney disease, work status, cognitive function, quality of social interaction, sleep quality, social support, sexual function, dialysis staff encouragement, and patient satisfaction. The eight SF-36 areas are physical functioning, role-physical, bodily pain, general health, social functioning, role-emotional, mental health, and vitality. The physical component summary (PCS) includes physical functioning, role-physical, bodily pain, and general health, while the mental component summary (MCS) includes social functioning, role-emotional, mental health, and vitality. The scoring criteria and methods for the KDQOL-SF 1.3 follow those reported by Hays et al. (1994). The total score range for the scale is 0–100, with higher scores indicative of better QOL. The Cronbach’s α coefficient of the KDQOL-SF 1.3 in this study was .851. The scale also earned a Kaiser–Meyer–Olkin score of .860, and an approximate χ^2^ score of 1,470.207 (*p* < .001) in Bartlett’s test of sphericity. These results support the good reliability and validity of this scale.

### Evaluation of Social Support

The Social Support Rating Scale (SSRS) was used in this study to assess the level of social support. Originally compiled by Xiao (1994), the SSRS has been widely used in related research. This scale includes three dimensions, namely objective support, emotional support, and utilization of social support, with higher dimension and total scale scores indicative of greater social support. In this study, the Cronbach’s α coefficient of the SSRS was .806, with a Kaiser–Meyer–Olkin measure of .786, and an approximate χ^2^ of 806.872 (*p* < .001) in Bartlett’s test of sphericity. These results support the good reliability and validity of this scale.

### Data Collection and Data Analysis

The 3-year follow-up investigative approach used in this study was designed to determine the effects of social support and quality of life on all-cause mortality in patients undergoing MHD. The research objectives were, first, to identify significant differences in social support and QOL scores between the deceased and surviving patient groups and, second, to analyze the predictors of all-cause mortality using Cox regression analysis.

#### Data collection

The tools used in data collection included a sociodemographic datasheet, the KDQOL-SF 1.3, and the SSRS.

#### Data analyses

Measurement data were expressed as mean ± standard deviation, while categorical data were expressed as percentages. Kolmogorov–Smirnov tests were used to perform normality tests on the measurement data, and, based on the results, *t* tests or Mann–Whitney tests were used to compare the between-group differences in measurement data. χ^2^ tests were used to compare between-group differences in enumeration data. The hazard ratio (HR) and 95% confidence interval of QOL and social support for overall mortality were calculated using Cox analysis. Multivariable Cox analysis was used after adjusting for age, educational status, employment status, primary renal diagnosis, and vascular access status. The level of significance used was .05, and all analyses were conducted using SPSS 16.0 (SPSS, Inc., Chicago, IL, USA).

### Ethical Considerations

All procedures involving human investigators in this study met ethical requirements and complied with the terms of the 1964 Helsinki Declaration as well as relevant ethical standards. All information was obtained with the informed consent of the participants. Participation in this study was wholly voluntary, and all participants’ identifying details were anonymized. This study was approved by the Medical Ethics Review Committee of Xi’an Medical University (XYLS2024220).

## Results

### Basic Characteristics

Initially, 286 participants were enrolled and completed the questionnaire at baseline. Over the 3-year follow-up, participants who transferred to another hospital (*n* = 31), received kidney transplantation (*n* = 6), or terminated their dialysis (*n* = 13) were excluded, leaving a valid sample of 236 participants to be tracked to the final outcome.

At the 3-year follow-up, 73 of the 236 participants had died (mortality rate: 30.93%). The mean age of participants was 58.21±15.83 years. Two-thirds (66.9%; *n* =158) were men, 64.8% had a high school or lower level of education, and only 16.9% were employed. Diabetic nephropathy was the most prevalent end-stage renal disease diagnosis (39.8%), and two-thirds (69.1%) used autologous arteriovenous fistula for vascular access (Table 1).

**Table 1:** Between-Group Comparison of Baseline General Characteristics

Characteristic	Total (*n* = 236)	Deceased Patients (*n* = 73)	Surviving Patients (*n* = 163)	*t/Z/*χ^2^	*p*
	*n* (%)	*n* (%)	*n* (%)		
Age (year, *M* and *SD*)	58.21±15.83	66.04±12.63	54.07±15.15	−5.445	<.001
Gender				0.001	.970
Male	158 (66.9)	49 (31.0)	109 (69.0)		
Female	78 (33.1)	24 (30.8)	54 (69.2)		
Educational level				3.874	.049
High school and below	153 (64.8)	54 (74.0)	99 (60.7)		
College and above	83 (35.2)	19 (26.0)	64 (39.3)		
Marital status				1.666	.197
Married	154 (65.3)	52 (71.8)	102 (62.6)		
Other (single, divorced, widowed)	82 (34.7)	21 (28.8)	61 (37.4)		
Employment status				12.132	.002
Working	40 (16.9)	8 (11.0)	32 (19.6)		
Retired	82 (34.7)	37 (50.7)	45 (27.6)		
Other (unemployed students)	114 (48.3)	28 (38.4)	86 (52.8)		
Primary renal diagnosis				16.522	.001
Glomerulonephritis	47 (19.9)	7 (9.6)	40 (24.5)		
Diabetic nephropathy	94 (39.8)	42 (57.5)	52 (31.9)		
Hypertensive nephropathy	54 (22.9)	16 (21.9)	38 (23.3)		
Other diagnosis	41 (17.4)	8 (11.0)	33 (20.2)		
SBP (mm Hg, *M* and *SD*)	150.89±18.38	154.80±16.11	149.70±18.90	−1.899	.058
DBP (mm Hg, *M* and *SD*)	88.43±54.44	90.29±70.31	84.94±13.75	−1.730	.084
Vascular access type				3.826	.050
Autologous arteriovenous fistula	163 (69.1)	44 (60.3)	119 (73.0)		
Deep vein catheterization	73 (30.9)	29 (39.7)	44 (27.0)		

*Note.* SBP = systolic blood pressure; DBP = diastolic blood pressure.

To investigate the impact of social support and QOL on prognosis, the participants were divided into two groups, namely deceased patients (*n* = 73) and surviving patients (*n* = 163), based on whether they had died over the 3-year follow-up period to examine differences in demographic and medical characteristics, social support, and QOL. The results of the comparison are presented in the following sections.

### Between-Group Comparison of General Characteristics

In terms of general characteristics, no significant between-group differences were found for gender, marriage, or blood pressure. The deceased patient group tended to be older (*Z* =-5.445, *p* < .001), have a lower level of education (*Z* = 3.874, *p* = .049), be currently unemployed (*Z* = 12.132, *p* = .002), have diabetic nephropathy as their primary disease (*Z* = 16.522, *p* = .001), and to have deep vein catheterization (*Z* = 3.826, *p* = .05; Table 1).

### Between-Group Comparison of Quality of Life

The average scores for the participants on the PCS, MCS, and SF-36 were 48.87±18.00, 62.92±17.53, and 56.63±15.03, respectively. Because questions regarding sexual activity are generally avoided in Chinese cultural settings, related questions were not included on the scales provided to the participants. Thus, between-group differences in the sexual dimensions were not examined. In this study, the scores of two indices in the physical component summary (physical functioning and role-physical), three indices in the mental component summary (social functioning, mental health, and vitality), and two indices in the kidney disease domain (effects of kidney disease and patient satisfaction) showed significant intergroup differences. Also, the surviving patient group earned higher mean PCS (*Z* = −3.794, *p* < .001), MCS (*Z* = −2.784, *p* = .005), and SF-36 (*Z* = −3.776, *p* < .001) scores than the deceased patient group (Table 2).

**Table 2:** Comparison of Baseline Quality of Life Between the Two Groups

Characteristic	Total (*n* = 236)	Deceased Patients (*n* = 73)	Surviving Patients (*n* = 163)	*Z*	*p*
Physical functioning	56.69±29.72	43.77±33.08	62.48±26.19	−3.937	<.001
Role-physical	30.30±38.71	20.89±34.11	34.51±39.99	−2.859	.004
Bodily pain	69.29±23.34	64.82±25.37	71.29±22.16	−1.493	.136
General health	47.48±19.92	44.38±19.63	48.87±19.96	−1.631	.103
Social functioning	62.24±23.95	54.97±26.74	65.49±21.91	−2.746	.006
Mental health	68.75±17.13	65.48±16.28	70.21±17.33	−2.279	.023
Role-emotional	59.04±46.00	54.34±46.32	61.15±45.85	−1.177	.239
Vitality	63.06±17.51	57.87±16.83	65.38±17.36	−3.370	.001
Physical component summary	48.87±18.00	42.36±17.29	51.80±17.58	−3.794	<.001
Mental component summary	62.92±17.53	58.33±16.55	64.97±17.62	−2.784	.005
SF-36	56.63±15.03	51.39±14.04	58.98±14.90	−3.776	<.001
Symptom/problem	75.03±15.94	72.72±18.24	76.07±14.73	−1.081	.280
Effects of kidney disease	46.30±20.26	41.50±20.53	48.45±19.82	−2.280	.023
Burden of kidney disease	31.65±19.79	29.45±18.00	32.63±20.52	−0.907	.365
Work status	43.41±26.20	43.63±24.72	43.31±26.91	−0.230	.818
Cognitive function	76.95±20.67	75.18±21.79	77.74±20.17	−0.638	.523
Quality of social interaction	69.80±17.71	67.85±17.51	70.67±17.79	−1.033	.302
Sleep quality	63.31±19.84	63.11±19.98	63.39±19.84	−0.117	.907
Social support	75.98±22.23	75.11±19.27	76.38±23.49	−1.040	.299
Dialysis staff encouragement	91.79±14.66	92.81±11.95	91.33±15.73	−0.378	.705
Patient satisfaction	73.79±20.50	78.76±16.25	71.57±21.82	−2.139	.032

*Note.* SF-36 = 36-item short-form health survey.

### Comparison of Social Support Differences Between the Two Groups

The average total SSRS score was 36.51 ± 8.03, while average scores for the three dimensions of objective support, emotional support, and utilization of social support were 8.76 ± 3.06, 20.84 ± 5.44, and 6.70 ± 2.01, respectively.

In terms of intergroup differences, no significant differences were found for objective support or emotional support. However, compared with the surviving patient group, the deceased patient group earned significantly lower mean scores for utilization of social support (*Z* = −2.497, *p* = .013) and the overall SSRS score (*Z* = −2.031, *p* = .042; Table 3).

**Table 3:** Comparison of Baseline Social Support Between the Two Groups

Characteristic	Total (*n* =236)	Deceased Patients (*n* =73)	Surviving Patients (*n* =163)	*Z*	*p*
Objective support	8.76±3.06	8.29±2.74	8.97±3.17	−1.261	.207
Emotional support	20.84±5.44	20.13±4.97	21.16±5.62	−1.245	.213
Utilization of social support	6.70±2.01	6.20±1.89	6.93±2.02	−2.497	.013
Social Support Rating Scale	36.51±8.03	34.72±7.45	37.32±8.18	−2.031	.042

### Multiple Factors Cox Regression

The results for the Cox proportional hazard models are shown in Table 4. After adjusting for age, education, employment, primary renal diagnosis, and vascular access, SF-36 (*p* = .041, HR = 0.983) and SSRS (*p* = .013, HR = 0.962) were found to be significantly associated with all-cause mortality. The HR values of the SSRS and SF-36 were both <1, indicating that higher social support and QOL scores are associated with a higher risk of mortality. The HR of SF-36 was 0.983, indicating that every 1-point increase in the SF-36 score decreased mortality risk by 1.7%, while the HR of SSRS was 0.962, indicating that every 1-point increase in the SSRS score decreased mortality risk by 3.8%. Moreover, the HR of age was >1, indicating that older age was associated with a higher risk of mortality.

**Table 4:** Relative Risk of Death According to the Cox Regression Analysis Models

Variable	Coefficient	Wald χ^2^	*p*	HR	95% CI of the HR
Age	0.043	23.833	<.001	1.044	[1.026, 1.062]
SF-36	−0.017	4.183	.041	0.983	[0.967, 0.999]
SSRS	−0.039	6.112	.013	0.962	[0.932, 0.992]

*Note.* HR = hazard ratio; SF-36 = 36-item short-form health survey; SSRS = Social Support Rating Scale.

## Discussion

The findings of this research revealed that those participants whose primary renal disease was diabetic nephropathy faced a higher risk of mortality, which is similar to the finding of Sitjar-Suñer et al. (2022). Previous studies have shown diabetes to increase the incidence of CKD-related renal function decline (Shanmukham et al., 2022). Other studies have found diabetes to be a predictor of mortality in patients with renal insufficiency (Klinger & Madziarska, 2019). While the results of the Cox regression analysis in this study did not show diabetes to directly influence mortality risk, diabetes may indirectly affect this risk. Diabetes-related complications such as diabetic foot and diabetic retinopathy may seriously affect patient vision and mobility, thus influencing their work, everyday activities, and social interactions and reducing their QOL. The findings of prior studies indicate that self-perceived physical and mental health in patients with diabetes receiving MHD has decreased (Gumprecht et al., 2010; Osthus et al., 2012).

Based on the findings presented in Tables 3 and 4, indicating social support is associated with mortality in patients undergoing MHD, good social support should be provided to patients undergoing hemodialysis to improve their prognosis. Due to frequent and regular dialysis, the patient’s social activities are significantly reduced. As a result, their entertainment and leisure time, and outlets to relieve depression and pain, are significantly reduced. Social support from families and hospitals is very important for these patients. Social support, as a positive predictor of mortality in patients on MHD, represents a significant, modifiable risk factor (Erickson et al., 2024). Good social support can improve depression, enhance quality of life, increase treatment compliance, and directly improve immune function in patients (Erickson et al., 2024). Social support from family, friends, colleagues, and other important people can have a positive effect (Hoang et al., 2022).

Patients must go to the hospital 2–3 times per week to receive MHD treatments lasting 4 hours each. Often, the relationship between patients and medical staff is like family. Medical staff manage the dialysis process of patients, handle various dialysis issues, and provide patients with professional support and encouragement, which is beneficial to their treatment and improves their prognosis (Ma et al., 2022). The patients trust medical staff and are willing to accept and follow their instructions (Ma et al., 2022). The social support of professional medical knowledge received from medical institution staff can further enhance the confidence of patients on dialysis. Related studies have shown that patients on dialysis who obtain more knowledge about kidney disease and dialysis from medical staff hold stronger treatment beliefs about their disease. When encountering negative events related to the disease, they can also make rational judgments and perceptions, and their sense of helplessness is lower (Xie et al., 2023). Patients not only receive professional medical knowledge from medical staff but also express their doubts and concerns to them. Medical staff can patiently comfort patients, providing them with greater social support (Merner et al., 2023). Support and encouragement from medical staff have been shown to be effective in improving well-being, information transfer, self-efficacy, and decision-making in patients with CKD. Also, patients receiving hemodialysis are grateful for the help and encouragement provided by medical staff, which improves the relationship between patients and medical staff and patient treatment efficacy. For medical staff, effective communication with patients, carefully designed nursing procedures, and continuity of care can promote good relationships between staff and patients (Jones et al., 2021), helping staff and patients communicate on renal treatments, especially hemodialysis, and improving treatment efficacy (Jones et al., 2021). Given the importance of knowledge about kidney disease and dialysis treatment for patients, medical staff should discuss disease and dialysis treatment knowledge regularly with patients to allow them to have a deeper understanding of relevant knowledge (Allen et al., 2022). Furthermore, it is necessary to build patient social support networks that include medical staff, family, friends, colleagues, and other important people to ensure social support plays a crucial role as an important external resource, relieving patient stress, reducing helplessness, and enhancing confidence in controlling a disease prognosis (Xie et al., 2023).

A correlation was identified in this study between QOL and prognoses in patients on hemodialysis. The SF-36 score was identified as a predictor of patient mortality, with higher QOL associated with lower mortality risk. Kim and Lee (2023) also reached the same conclusion using the World Health Organization Quality of Life Questionnaire-Brief version, showing the physiological and psychological scores of the deceased group to be lower than those of the surviving group. A significant correlation exists between lower QOL and higher mortality rate (J. Lee et al., 2020). Lee found a correlation among overall and physiological domain QOL scores and mortality, but no significant correlation with the QOL psychological domain (J. Lee et al., 2020). However, in another study, SF-36 scores measured twice at 6-month intervals showed the mortality rate 1 year after the second test was 1.33-fold higher when the score for the psychological domain had decreased by at least 5 points over the same time period (Liebman et al., 2016). Different from Liebman and most other studies, this research was designed to track prognoses over a significantly longer period (3 years).

Because PCS, MCS, and SF-36 scores are all calculated based on their respective dimension scores, to prevent mutual influence between variables, only PCS, MCS, and SF-36 scores were included in the final Cox regression analysis. The results show that only the SF-36 score, which reflects the overall psychological and physiological situation, was significantly related to mortality risk. Brito and colleagues reported a significant association between the physiological domain and mortality risk after 1 year of follow-up, and significant associations between both the physiological domain and SF-36 score and mortality risk after 5- and 9-year follow-ups after dialysis. However, no association was found between the psychological domain and mortality (Brito et al., 2020). Similarly, another study also found the physical health domain to have the greatest impact on mortality risk in patients on hemodialysis (Kim & Lee, 2023). The PCS score is beneficial in evaluating patient health status and providing a reference for treatment plans, improving overall QOL and health status in patients on MHD. In addition, psychological function has been shown to be a predictor of mortality risk in patients. A survey of patients in the Dialysis Outcomes and Practice Patterns Study cohort under the age of 60 showed a correlation between MCS score and adjusted all-cause mortality (Mapes et al., 2003). Other studies have confirmed a significant negative correlation between MCS score and all-cause mortality in elderly patients on dialysis, with higher MCS scores indicating lower all-cause mortality (Lacson et al., 2010; Lowrie et al., 2003). Improving the psychological condition of patients can improve their prognosis, with related studies showing that increasing handgrip strength in patients with CKD can enhance their self-care ability, further improve their depressive status, and ultimately improve their QOL (S.-Y. Lee et al., 2024). Health literacy education for patients with stage 3–4 CKD can improve their health literacy, depression status, and renal functioning (Huang et al., 2024).

There are several strengths of this study. First, the prospective design used minimizes the effect of recall bias on outcomes. Second, few studies in the literature have analyzed the impact of social support on patient mortality risk. In this study, the impacts of social support and QOL on all-cause mortality in patients were analyzed comprehensively.

This study was also affected by several limitations. First, despite its 3-year follow-up design, the observational nature of this study disallows confirming causal relationships between social support and quality of life, respectively, and all-cause mortality. Second, due to time limitations, patient survival was only tracked for 3 years. However, the prognoses of these patients will continue to be tracked by the researchers. Finally, this study only involved patients on hemodialysis. Further research is needed to investigate the similarities/differences in patients on peritoneal dialysis.

In the future, intervention studies should be conducted to analyze whether patient prognoses may be improved and whether the mortality rate is decreased after enhancing their social support and quality of life factors, including psychological and physiological factors. Also, social support and quality of life were only evaluated at baseline. Future research should consider the changes in these variables over time.

### Conclusions

The findings of this study support the presence of a significant association between social support and QOL, respectively, and all-cause mortality in patients on MHD. The results confirm the importance of enhancing QOL and social support in improving the prognoses of patients on hemodialysis. Targeted intervention measures can help strengthen patient care and encouragement by establishing a social network of medical staff, family, friends, colleagues, and other important individuals. By improving patients’ physiological and psychological conditions, their overall quality of life can be enhanced, thereby improving their prognosis.
